# Dynamic versus fixed cerebral perfusion pressure targets in paediatric traumatic brain injury: a STARSHIP analysis

**DOI:** 10.1016/j.eclinm.2025.103370

**Published:** 2025-07-17

**Authors:** C.A. Smith, S.Y. Bögli, M.M. Placek, M. Cabeleira, D. White, E. Daubney, A. Young, E. Beqiri, R. Kayani, R. O'Donnell, N. Pathan, S. Watson, A. Maw, M. Garnett, H.K. Kanthimathinathan, H. Bangalore, S. Sundararajan, G. Subramanian, D. Raffaj, S. Lampariello, A. Sarfatti, A. Mayer, O. Ross, M. Czosnyka, P.J. Hutchinson, P. Smielewski, S. Agrawal, STARSHIP Study Team, Shruti Agrawal, Peter Smielewski, Peter J. Hutchinson, Stefan Yu Bögli, Claudia A. Smith, Carly Tooke, Caroline Payne, Holly Belfield, Amisha Mistry, Collette Spencer, Claire Jennings, Lara Bunni, Laura Anderson, Emily Morgan, Melanie James, Rebecca Beckley, Tahnima Khatun, Hafiza Khatun, Olivia Nugent, Richard Aldridge, Ruth Morgan, Julie Morcombe, Martin Quinton, Catherine Postlethwaite, Jenny Pond, Jessica Cutler, Caitlin Oxford, Marek Czosnyka, Michal Placek, Manuel Cabaleira, Deborah White, Esther Daubney, Adam Young, Erta Beqiri, Riaz Kayani, Roddy O'Donnell, Nazima Pathan, Suzanna Watson, Anna Maw, Matthew Garnett, Hari Krishnan Kanthimathinathan, Harish Bangalore, Santosh Sundararajan, Gayathri Subramanian, Dusan Raffaj, Simona Lampariello, Avishay Sarfatti, Anton Mayer, Oliver Ross

**Affiliations:** a Department of Clinical Neurosciences, University of Cambridge, Cambridge, UK; b Department of Mechanical Engineering, University College London, London, UK; c Department of Paediatrics, University of Cambridge, Cambridge, UK; d Paediatric Intensive Care, Cambridge University Hospitals, Cambridge, UK; e Paediatric Neuropsychology, Cambridge and Peterborough NHS Foundation Trust, Cambridge, UK; f Paediatric Intensive Care, Birmingham Children's Hospital, Birmingham, UK; g Paediatric Intensive Care, Great Ormond Street Hospital, London, UK; h Paediatric Intensive Care, Leeds Children's Hospital, Leeds, UK; i Paediatric Intensive Care, Manchester Children's Hospital, Manchester, UK; j Paediatric Intensive Care, Nottingham Children's Hospital, Nottingham, UK; k Paediatric Intensive Care, Oxford University Hospitals, Oxford, UK; l Paediatric Intensive Care, Royal London Hospital, London, UK; m Paediatric Intensive Care, Sheffield Children's Hospital, Sheffield, UK; n Paediatric Intensive Care, Southampton General Hospital, Southampton, UK; o Institute of Electronic Systems, Warsaw University of Technology, Poland; p Department for Neurology and Neurocritical Care Unit, University Hospital and University of Zurich, Zurich, Switzerland

**Keywords:** Paediatric traumatic brain injury, Multimodality neuromonitoring, Intensive care management, Cerebral perfusion pressure, Cerebrovascular autoregulation

## Abstract

**Background:**

Cerebral perfusion pressure (CPP) represents a key target for intensive care management of paediatric traumatic brain injury (TBI) patients. Current guidelines recommend a CPP target within the range of 40–50 mmHg but emphasise that these may depend on patient age and the state of cerebrovascular autoregulation. In this analysis, we aimed to compare the fixed targets proposed by the Brain Trauma Foundation to autoregulation-based targets CPPopt (optimal CPP) and LLA (Lower Limit of Autoregulation).

**Methods:**

Data were acquired from the STARSHIP study (a prospective, multicentre, observational, research study which enrolled 135 children (median age 96 months (interquartile range 26–152 months)) with TBI between July 2018 and March 2023 across 10 paediatric intensive care units in the UK). In this secondary analysis the dose or percentage time spent below a fixed CPP target of 50 mmHg or CPPopt or LLA (assessed continuously on a minute-by-minute basis and derived by fitting a curve to the relationship between CPP and pressure reactivity index values, as previously described) was compared by outcome using univariable and multivariable methods. ClinicalTrials.gov registration–NCT0688462.

**Findings:**

When assessed within ordinal analyses (to account for differences in baseline severity), both hourly dose and percentage time spent below LLA (odds ratio 1.01 [95% CI 1.00–1.02], p = 0.017 and 1.05 [95% CI 1.01–1.08], p = 0.008 respectively) were independently associated with worse outcomes. LLA displayed a dynamic time-trend increasing over time in patients with unfavourable outcome (n = 44, p = 0.003). Overall, LLA exceeded 50 mmHg for more than 45% of the monitoring period across all patients, and for over 35% of the time in the youngest cohort (0–2 years).

**Interpretation:**

Dynamic autoregulation monitoring based on LLA was associated with outcomes in paediatric TBI with higher LLA values observed in individuals experiencing unfavourable outcomes. Our findings indicate that the current fixed CPP threshold of 40–50 mmHg may be too low–highlighting the need for further investigation into autoregulation-guided CPP targets. Whether personalised management based on autoregulatory-informed thresholds offers advantages over guideline-based targets remains to be determined and should be investigated in future prospective interventional studies.

**Funding:**

10.13039/501100000317Action Medical Research for Childrens' Charity and 10.13039/501100002927Addenbrookes Charitable Trust (UK Grant number–GN2609).

**Research in context**

**Evidence before this study**

Cerebral perfusion pressure (CPP) is widely acknowledged as an important therapeutic target in the management of paediatric traumatic brain injury (TBI). Current Brain Trauma Foundation (BTF) guidelines advocate a fixed lower CPP threshold of 40–50 mmHg—presumed to represent the lower limit of autoregulation (LLA)—based primarily on retrospective analyses. Whether cerebrovascular autoregulation informed CPP targets are more closely associated to outcome compared to the fixed thresholds, remains debated.

**Added value of this study**

Using data obtained from the STARSHIP study (“Studying Trends in AutoRegulation in Severe Head Injury in Paediatrics”) and employing rigorous statistical methods, including ordinal analyses, this investigation demonstrates that CPP deviations below the LLA have potential to be explored as prognostic markers. In contrast, deviations from the fixed CPP thresholds recommended by the BTF did not show the same predictive power in this study. The LLA varied both with patient age and dynamically during the course of TBI, with higher LLA values observed in individuals who ultimately experience unfavourable outcomes.

**Implications of all the available evidence**

These findings suggest that the current recommended fixed lower CPP limit of 40–50 mmHg is likely too low for most children, regardless of age. The evidence generated in this study offers a basis for further external validation and will inform Phase II trials aimed at refining CPP targets in paediatric TBI.

## Introduction

Cerebral perfusion pressure (CPP) is a critical metric in the management of paediatric traumatic brain injury (TBI). CPP is the driving pressure for blood flow through the cerebrovascular bed and is calculated as the mathematical difference between arterial blood pressure (ABP) and intracranial pressure (ICP). CPP management is pivotal for sustaining a sufficient cerebral blood flow and consequently for sustaining brain metabolism and function. Current Brain Trauma Foundation (BTF) guidelines recommend CPPs above a fixed minimum (i.e., lower limit) of 40–50 mmHg (with higher minimum values for older children).^1^ The brain has an innate mechanism to control cerebral blood flow, called cerebrovascular autoregulation.^2^ This dynamic homoeostatic mechanism adjusts arteriolar diameters depending on CPP. When cerebrovascular autoregulation is intact, the brain can–within certain limits–maintain stable cerebral blood flow despite changes in CPP. However, in paediatric TBI, this mechanism is often impaired, making CPP management critical.

The state of cerebrovascular autoregulation can be assessed using the pressure reactivity index (PRx), a widely used index in adult neurocritical care.^3^ PRx is a correlation coefficient which quantifies the relationship between slow changes (averages over 10 s) in ABP and ICP. Studying Trends in AutoRegulation in Severe Head Injury in Paediatrics (STARSHIP)–the largest multicentre prospective study on PRx in paediatric TBI–demonstrated the validity of PRx for prognostication.^4^ PRx can provide valuable insights in understanding CPP limits and targets in TBI. In adults, when PRx is plotted against CPP, it often forms a U-shaped curve, with the lowest point representing the optimal CPP (CPPopt).^5^^,^^6^ CPPopt is the CPP value associated with the lowest PRx, indicating the ‘best’ autoregulatory function, and adult research has shown that targeting the CPPopt is safe and feasible, and may under certain circumstances lead to PRx improvement.^7^ The Lower Limit of Autoregulation (LLA) is the CPP value, below which cerebrovascular autoregulation fails. Deviation in CPP from both CPPopt, and LLA, have been associated with death and worse outcomes following adult TBI.^5^^,^^6^^,^^8^^,^^9^ However, it remains to be established which of the two targets–CPPopt or LLA–is more appropriate for prognostication and the clinical management of TBI. It is important to note that while early analyses of both CPPopt and LLA were conducted retrospectively using the full monitoring data of each patient,^5^ more recent advancements permit dynamic estimation of these targets in real-time at the bed-side.^10^, ^11^, ^12^, ^13^ This is achieved through repeated calculation utilising moving data buffers of 2–8 h, enabling the acquisition of minute-by-minute trends for both CPPopt and LLA. Although no interventional studies have demonstrated a definitive benefit of targeting CPPopt at the bedside to date, this approach has been shown to be both safe and feasible.^7^ Conceptually, when CPP is maintained at CPPopt, the autoregulatory system operates “optimally”, achieving stable cerebral blood flow despite fluctuations in CPP. In contrast, the LLA represents a critical “safety” threshold–if CPP falls below the LLA, cerebral blood flow decreases linearly with further reductions in CPP.^14^ Investigations in children are limited to relatively small retrospective studies with up to 67 patients.^15^, ^16^, ^17^, ^18^ These almost exclusively asses CPPopt, with only one study assessing both CPPopt and LLA.^15^

Although preliminary studies suggest that autoregulation-informed CPP targets may hold promise for improving prognostication following paediatric TBI, the identification of optimal CPP thresholds in this population remains critically understudied. Accordingly, one of the secondary objectives of the initial STARSHIP study analysis plan^19^ was to characterise optimal CPP thresholds in paediatric TBI. Leveraging the STARSHIP research database, this study pursues two primary aims: first, to compare autoregulation-informed CPP targets–specifically CPPopt and the LLA–to identify the superior prognostic metric; and second, to assess the performance of autoregulation informed targets relative to the fixed CPP thresholds recommended by the BTF guidelines.

## Methods

**Fig. 1:**
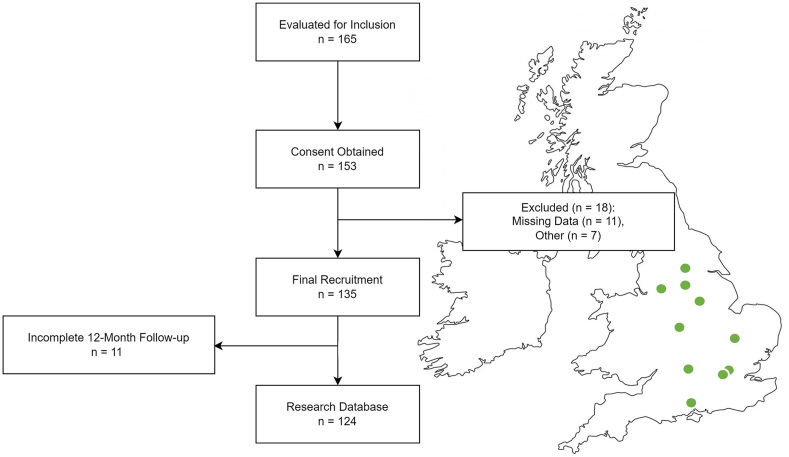
**Flowchart of participant recruitment and data inclusion.** A total of 165 individuals were evaluated for inclusion, of whom 153 provided consent. Eighteen participants were excluded due to missing data (n = 11) or other reasons (n = 7), resulting in a final recruited cohort of 135. Twelve-month follow-up was incomplete for 11 individuals, yielding a final sample of 124 for the research database. The map illustrates the geographical distribution of participating centres across the United Kingdom (green circles).

STARSHIP represents the first prospective multi-centre observational study to assess cerebrovascular autoregulation in paediatric head injury.^19^ Patients were enrolled across 10 paediatric intensive care units within the UK (9 of which are Children's Major Trauma Centres, representing centres that provide specialised trauma care with an interdisciplinary team of physicians). Fig. 1 presents the inclusion and exclusion flowchart alongside a geographical representation of the participating centres. Notably, the present study represents a secondary analysis of the STARSHIP data and does not follow a pre-specified analytical plan. The sample size was determined only for the analyses described in the primary exploration.^4^

The inclusion criteria for the STARSHIP study were as follows: age ≤16 years, admission to intensive care with TBI (defined as a Glasgow Coma Scale score ≤8 with TBI-related pathology confirmed by neuroimaging), and invasive monitoring of ICP and ABP. No exclusion criteria were applied. The management across the different centres, which includes ICP and CPP management, largely aligns with the BTF guidelines.^1^ Similarly, the initiation of invasive monitoring largely follows the recommendations provided by the BTF; however, the ultimate decision rests with the treating physician. Clear indications for invasive monitoring include patients for whom sedation interruption poses safety concerns and patients following the evacuation of an acute haematoma who remain at high risk for intracranial hypertension. While treating clinicians were not blinded to the bedside data, STARSHIP was a purely observational trial, and decisions were not informed by study data (including PRx, CPPopt or LLA).

### Ethics

Informed consent for the study, and additional consent for follow-up was obtained by patient's parents/guardians. STARSHIP was approved by the Health Research Authority, South West-Central Bristol Research Ethics Committee (Ref: 18/SW/0053, 23/SW/0011).

### Data acquisition, curation, and processing

Data acquisition, curation, and processing were performed as previously described.^4^^,^^19^ In all patients, ICP was measured using intraparenchymal probes (Codman ICP MicroSensor, Codman & Shurtleff, Raynham, Massachusetts) and ABP was measured using a radial/femoral arterial line (Baxter Healthcare, Deerfield, Illinois) zeroed at the level of the right atrium. Real time high resolution (250 Hz) physiological monitoring was performed in the ICM+ software (Cambridge Enterprise, Cambridge, UK), as well as all cleaning, processing, and calculation of metrics. Large artefacts relating to non-physiological disturbances to the physiological waveform, including periods of signal disconnections or flat lines, were removed prior to analysis. Further, automated artefact markup was applied which included lack of pulsatility, and non-physiological minima/maxima. Post cleaning, minute by minute averages were calculated for each physiological signal. In addition to physiological parameters, clinical and demographic data were collected.

### Outcome data

Patient outcomes were evaluated using two measures: (1) mortality at the time of discharge and (2) functional outcomes at 12 months, assessed using the Glasgow Outcome Scale-Extended Paediatric Revision (GOS-E Peds). The GOS-E Peds assessments were conducted either in person during outpatient visits or via telephone interviews. Mortality data at discharge was available for all 135 patients, while 12-month GOS-E Peds scores were recorded for 124 patients. The GOS-E Peds scale ranges from 1 (upper good recovery) to 8 (death). For outcome analysis, the 12-month GOS-E Peds scores were categorised as either favourable (scores 1–4) or unfavourable (scores 5–8).

### Calculation of CPP, CPPopt, LLA, and dose metrics

**Fig. 2:**
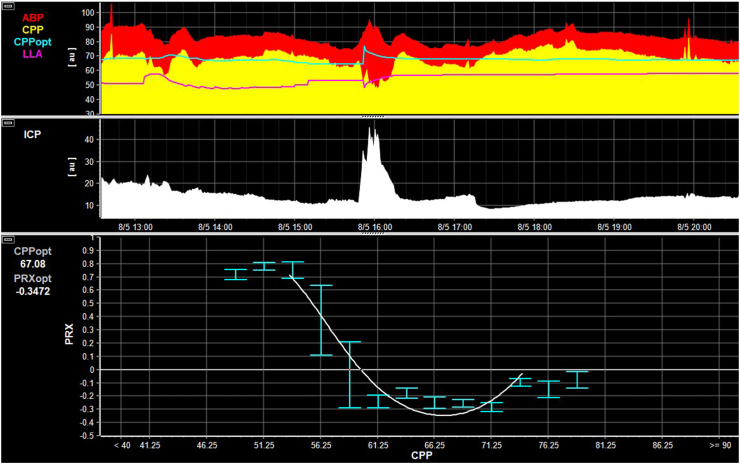
**Example of optimal CPP (CPPopt) and lower limit of autoregulation (LLA) calculation.** An example 8 h section of multimodality monitoring data is shown. On top the minute-by-minute ABP and CPP time trends are shown. Superimposed are the dynamic CPPopt (blue) and LLA (pink) time-trends which are estimated every minute considering the last 2–8 h of data. Below, the minute-by-minute ICP are shown. On the bottom, the overall relationship between CPP and PRx (considering the full data) is shown. When considering the overall data, CPPopt is around 67 mmHg, while the LLA is around 57 mmHg. The example also allows to visualise the different derived metrics. Specifically, the overall dose represents the area under the curve, calculated as the cumulative deviation from the target CPP when actual CPP falls below this threshold. The hourly dose is defined as the overall dose divided by the total duration of monitoring, whereas percentage time refers to the proportion of monitoring time during which the actual CPP remains below the predefined target.

CPPopt was calculated as the CPP corresponding to the lowest PRx value, determined by fitting a parabolic curve to 5-min median CPP and PRx values. CPP bins were set at 2.5 mmHg, with additional heuristic algorithmic details as previously described.^6^ The bin width was selected based on carefully cleaned data and visual inspection of CPPopt curves. Within the same curves, LLA was defined as the CPP where cerebrovascular autoregulation was already impaired (i.e., PRx = 0.2) and worsened with further decreases in CPP. The yield of CPPopt is the percentage of time CPPopt calculation is possible. In all cases, per patient means were calculated. Fig. 1 details the concept of CPPopt and LLA. To quantify the deviations from different personalised and fixed CPP targets, we quantified the deviations from CPPopt, LLA, or a fixed CPP target of 50 mmHg using the following metrics: (1) overall dose (in mmHg∗h), defined as the area under the curve below the CPP cutoff; (2) hourly dose (in mmHg), representing the total dose divided by hours of valid data; and (3) percentage time (ptime, in %), of valid data, during which CPP was below the target. These concepts are further illustrated in Fig. 2, which presents both the global assessments and the dynamic calculation methods.

### Statistics

Statistical analyses and figure preparation were performed using R studio software (version 4.4.1: https://www.r-project.org/–packages used: *dplyr*, *rstatix*, *gtsummary*, *MASS*, *pROC*, *ggplot2*, *lme4*, *gbmt*), and statistical significance was set at the 0.05 level. To account for multiple comparisons, the Benjamini–Hochberg procedure^20^ was applied limiting the proportion of false positives as appropriate.

To investigate the relationship between various CPPopt and LLA metrics and outcome, the variables were investigated with univariable analysis using Mann Whitney's (Wilcoxon rank) test for both mortality at discharge, and dichotomised outcome at 12 months post-ictus. The cohort median and interquartile range (IQR) are reported for the various metrics. Due to limited sample size in the non-survivor group (10 patients), multivariable regression was conducted on 124 patients with dichotomised 12-month outcomes as the endpoint. As CPPopt was not significantly different between outcome groups, only LLA was explored further. To allow for exploration of the ordinal range of the GOSE-P, a sliding dichotomy approach was performed to account for initial clinical severity in line with the previous description by Roozenbeek et al.^21^ In a first step, prognostic risk scores were calculated for each patient. Specifically, these were estimated by applying logistic regression including the covariates age, motor GCS, pupillary reactivity, ISS, Rotterdam score, pre-admission hypoxia, and pre-admission hypotension, and cardiac arrest (largely in line with the IMPACT score^22^). The number of covariates (as compared to the number of patients) does not affect the robustness of this methodology, and hence its use is supported by the literature.^23^^,^^24^ The resulting scores were then divided into 3 prognostic groups of roughly equal size corresponding to low, intermediate, and high likelihood of unfavourable outcome. For each prognostic group a separate cutoff was defined to dichotomise outcome into favourable and unfavourable groups, with the adjusted favourable outcome classified as: GOSE-Peds ≤ 2 for low, GOSE-Peds ≤ 4 for the group with intermediate, and GOSE-Peds ≤ 6 for the group with high likelihood of unfavourable outcome. The resulting baseline severity adjusted outcome variable was then assessed against the CPP metrics and ICP using logistic regression. Odds ratios (ORs) are reported. For all metrics bootstrapping was applied for internal validation and to acquire the 95% confidence intervals (CIs). In summary, this analysis evaluates whether the patients' outcome was poorer than expected, given the severity of the initial injury.

To further explore LLA, the temporal dynamics of LLA were visualised using locally estimated scatterplot smoothing, stratified by patient outcome. Subsequently, mixed-effects models were constructed to statistically assess differences in LLA trajectories by outcome, incorporating outcome as the dependent variable, LLA and monitoring day as fixed effects, and patient ID as a random effect to account for individual variability in baseline trajectories. Second, considering that LLA thresholds are inherently patient-specific and that younger patients likely require lower CPP targets,^1^ we explored age-related differences by plotting the distribution of hourly LLA values. These distributions were presented as percentages of available data and grouped according to patient age categories: 0–2 years, 2–8 years, and ≥8 years.

Lastly, we directly compared LLA derived metrics to CPP targets suggested by the BTF. Specifically, we first assessed their performance within univariable Receiver Operating Characteristic (ROC) curves, specifically looking at the area under the curve (AUC) and the associated 95% CI. Additionally, we explored whether significant monitoring-based metrics improved prognostic models that included readily available clinical parameters (represented by the prognostic risk scores described previously). The added value of these metrics was quantified using the continuous Net Reclassification Index,^25^ which measures the proportion of patients whose prognostic accuracy improves after incorporating these monitoring-derived metrics.

### Role of the funding source

The funders or the sponsor did not have any role in the collection, management, analysis, and interpretation of the data; preparation, review, or approval of the manuscript; or decision to submit the manuscript for publication, the right to veto publication or control the decision regarding which journal the manuscript was submitted.

## Results

135 children with paediatric TBI requiring invasive monitoring were included in this analysis; the STARSHIP cohort has been previously described.^4^ The patient characteristics are described in Table 1. The median age of patients was 96 months (IQR 26–152) with the majority being male (78%). Overall, 44% suffered an isolated TBI with high overall severity as assessed using the Injury Severity Score (ISS; median 29, IQR 25–45) and the Rotterdam score (median 3, IQR 2–3). Overall, 23 (17%) patients received a decompressive craniectomy, 27 (20%) received a haematoma evacuation, and 14 (10%) received an external ventricular drain for ICP management. Data coverage and the corresponding distribution by day are shown in Supplement A. Briefly, the median number of monitoring hours were 157 (IQR 88–279) for ABP and 153 (IQR 88–275) for ICP respectively. Monitoring largely started directly after intensive care admission. Median LLA and CPPopt for the cohort were 52.3 mmHg and 65.3 mmHg respectively. The CPPopt yield was 88%.

**Table 1:** Patient characteristics.^a^

Characteristic	N = 135
Sex (male)	105 (78%)
Age (months)	96 (26, 152)
Type of injury	
Blunt	130 (96%)
Penetrating	5 (4%)
Isolated TBI	60 (44%)
Mechanism of injury	
Road traffic accident	63 (47%)
Fall	27 (20%)
Bicycle accident	24 (18%)
Other	11 (8%)
Inflicted injury	10 (7%)
GCS total	6 (3, 8)
GCS motor	3 (1, 5)
Pupillary reactivity	
Both reactive	102 (80%)
One reactive	15 (12%)
None reactive	11 (9%)
Prehospital events	
Hypoxia	19 (14%)
Hypotension	26 (19%)
Cardiac arrest	7 (5%)
Marshall score	2 (2, 3)
Rotterdam score	3 (2, 3)
AIS head	5 (4, 5)
ISS	29 (25, 45)

Abbreviations: GCS = Glasgow coma scale, AIS = Abbreviated injury score, ISS= Injury severity score.

a Data shown as number (%) or median (interquartile range).

The univariable analysis is described in Table 2. Notably, no CPPopt metrics were associated with outcome in univariable analysis. Conversely, patients who suffered an unfavourable outcome at 12 months had a greater hourly dose of CPP below LLA (median 22 mmHg [IQR 13–51] vs. 55 mmHg [IQR 21–100], p = 0.04, for patients with favourable and unfavourable outcomes respectively). Similarly, these patients also spent a greater amount of time below LLA (9% [IQR 5–18] vs. 18% [IQR 8–27], p = 0.04, for patients with favourable and unfavourable outcomes respectively). The multivariable, sliding dichotomy based, analysis is described in Table 2 and in Supplement B. Supplement B describes clinical parameters stratified by the prognostic risk group. Overall, patients with a higher risk of unfavourable outcome had sustained a more severe injury (i.e., higher ISS and Rotterdam score, p < 0.001). The residual effect of the CPP parameters is presented in Table 2. Overall hourly dose of CPP below LLA (OR 1.12 [CI 1.03–1.24], p = 0.018) and percentage time with CPP below LLA (OR 1.25 [CI 1.07–1.49], p = 0.008) remained associated with outcome when added to multivariable logistic regression models with mean ICP for prediction of adjusted outcome categorisation (Table 3).

**Table 2:** Summary metrics of CPPopt and LLA, including dose variables, for the cohort, and by mortality and dichotomous outcome groups.^a^

Characteristic	Entire cohort	Discharge mortality			12 month dichotomised outcome		
	N = 135	Non-survivor, N = 10	Survivor, N = 125	p-value	Unfavourable, N = 44	Favourable, N = 80	p-value
CPPopt (mmHg)	65.3 (61.5, 68.4)	61.4 (56.6, 67.2)	65.5 (61.7, 68.4)	0.5	65.5 (59.5, 67.8)	64.9 (61.6, 68.2)	>0.9
CPPopt yield (%)	88 (81, 92)	64 (13, 91)	88 (81, 92)	0.3	87 (71, 91)	89 (84, 92)	0.2
Overall dose of CPP below CPPopt	6334 (2531, 14,168)	9502 (2926, 17,824)	6334 (2473, 13,497)	0.5	5280 (2531, 14,794)	6591 (2777, 11,703)	0.8
Hourly dose of CPP below CPPopt	90 (64, 142)	161 (114, 251)	90 (62, 140)	0.5	93 (66, 145)	89 (59, 131)	0.7
ptime with CPP below CPPopt (%)	29 (21, 39)	46 (33, 52)	28 (21, 38)	0.5	31 (24, 41)	27 (21, 38)	0.4
LLA (mmHg)	52.3 (48.9, 57.2)	53.7 (50.3, 58.2)	52.3 (48.9, 57.2)	0.7	55.0 (50.2, 57.4)	51.9 (48.3, 57.3)	0.2
Overall dose of CPP below LLA	1997 (752, 4600)	7319 (4,183, 8181)	1875 (644, 4247)	0.037	3885 (885, 7356)	1765 (772, 3655)	0.082
Hourly dose of CPP below LLA	29 (14, 69)	118 (89, 243)	26 (14, 62)	0.020	55 (21, 99)	22 (14, 51)	0.04
ptime with CPP below LLA (%)	11 (5, 22)	26 (20, 67)	10 (5, 21)	0.037	18 (8, 27)	9 (5, 18)	0.04

Abbreviations: CPPopt = optimal CPP, Δ = delta, CPP = cerebral perfusion pressure, ptime = percentage time, LLA = lower limit of autoregulation, N = number of patients.

a Data shown as median (interquartile range).

**Table 3:** Sliding dichotomy approach.

Characteristic	LLA metric		ICP	
	OR (95% CI)	p-value	OR (95% CI)	p-value
LLA (mmHg) + ICP	1.06 (0.99, 1.14)	0.1	1.20 (1.08, 1.36)	0.002
Overall dose^a^ of CPP below LLA + ICP	1.05 (0.97, 1.15)	0.2	1.15 (1.04, 1.30)	0.014
Hourly dose^a^ of CPP below LLA + ICP	1.12 (1.03, 1.24)	0.018	1.12 (1.00, 1.28)	0.062
ptime^a^ with CPP below LLA (%) + ICP	1.25 (1.07, 1.49)	0.008	1.14 (1.02, 1.30)	0.030

The results of the multivariable logistic regression considering LLA metrics and ICP for the prediction of the covariate adjusted outcome definition is shown using odds ratios and corresponding confidence intervals.

Abbreviations: ICP = intracranial pressure; ptime = percentage time, LLA = lower limit of autoregulation, N = number of patients.

a The derived odds ratios are displayed for every 1000 mmHg∗h dose, 10 mmHg hourly dose, and 5% ptime.

**Fig. 3:**
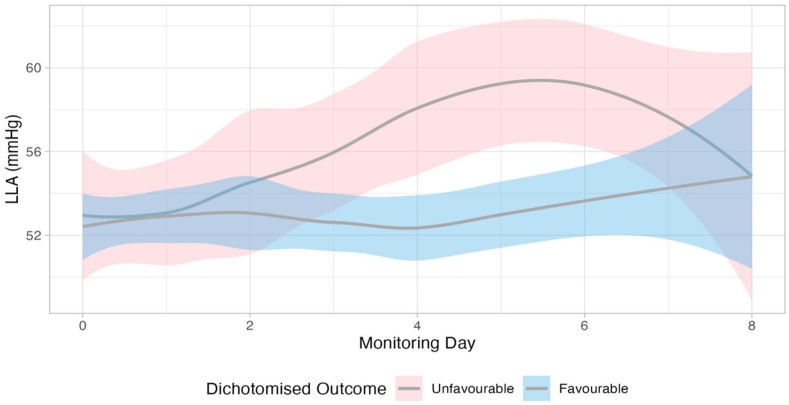
**LLA over time for unfavourable and favourable outcome groups.** When modelled using a linear mixed effects model including the patient as a random effect, there was a significant interaction between the level of LLA and day with a larger increase over time for patients with worse outcome (p = 0.003). Coloured ribbons represent the 95% confidence interval.

**Fig. 4:**
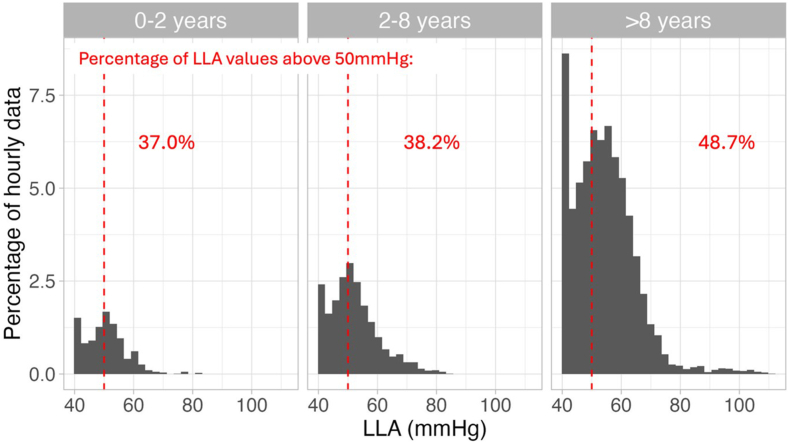
**The mean LLA distribution for each age group.** Current guidelines recommend a CPP target between 40 and 50 mmHg, and hence a red vertical 50 mmHg reference line is shown. For each age group, the percentage of LLAs above this 50 mmHg line is reported. The cohort's median LLA was 50 mmHg (IQR 45–54), 51 mmHg (IQR 46–56), 53 mmHg (IQR 47–60) for the patients aged 0–2, 2–8, and above 8 respectively (p < 0.001).

The temporal profile of LLA by outcome group is shown in Fig. 3. Patients with unfavourable outcomes consistently displayed higher LLA compared to patients with favourable outcomes. Noticeably, in patients with unfavourable outcomes, the LLA peaked at around 59 mmHg at day 5 from injury and subsequently decreased to the level of patients with favourable outcomes (around 55 mmHg) around day 8. Fig. 4 shows the distribution of hourly LLA values in the STARSHIP cohort, stratified by age group. All age groups had a relevant percentage of time, during which LLA was above the target of 50 mmHg CPP suggested by the BTF (37.0%, 38.2%, 48.7% for children aged 0–2 years, 2–8 years, and above 8 years).

**Fig. 5:**
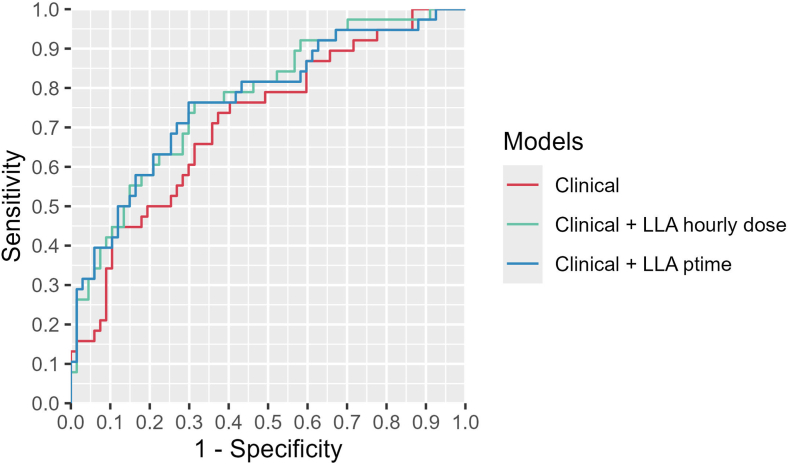
**The receiver operating characteristics curves show the predictive benefit of LLA derived measures, over and above clinical metrics.** The receiver operating characteristics curves for the different models is displayed, with models including only the clinical parameters (red), clinical parameters with the hourly dose of cerebral perfusion pressure below the lower limit of autoregulation (green), and clinical parameters with the percentage time of cerebral perfusion pressure below the lower limit of autoregulation (blue).

In the next step, we compared autoregulation informed CPP targets to the fixed target suggested by the BTF. When assessing the value of the different targets within univariable ROC curves for the prediction of unfavourable outcomes, the AUCs of dose and ptime below a fixed CPP target of 50 mmHg displayed low AUC with the corresponding CI crossing 0.5 suggesting no prognostic relevance (AUC of 0.57 [CI 0.46–0.68] for dose and 0.56 [CI 0.46–0.68] for ptime). Conversely both hourly dose (AUC of 0.70 [CI 0.51–0.73]) and ptime (AUC of 0.68 [CI 0.54–0.75]) below LLA displayed discriminatory value with a univariable accuracy of 0.68 for both hourly dose and ptime. Lastly, we assessed whether adding the different LLA metrics would improve the diagnostic performance compared to models including clinical parameters only. The receiver operating characteristics AUCs considering either only clinical parameters or clinical parameters + LLA hourly dose or clinical parameters + LLA ptime are shown in Fig. 5. The respective AUCs were 0.72 (CI 0.62–0.82) for clinical parameters only, 0.77 (CI 0.68–0.87) for clinical parameters and LLA hourly dose, and 0.77 (CI 0.67–0.87) for LLA and LLA ptime. The net reclassification index was 0.55 and 0.60 for the addition of dose and ptime representing an improvement in predictive performance when including the LLA metrics.

## Discussion

Using the largest prospective multicentre observational multimodality monitoring database of paediatric TBI to date, we characterised the associations between different CPP targets for prognostication. Specifically, we explored the fixed targets proposed by the BTF, and the autoregulation informed targets CPPopt and LLA. The main results showed the following: Deviations from CPPopt were not associated with outcome. Conversely, deviations from the LLA were associated with outcome, even after adjustment for relevant clinical prognostic parameters. The LLA changed dynamically after the initial injury and tended to increase in patients with unfavourable outcomes. Furthermore, with increasing age, the fixed CPP targets recommended by the BTF deviated increasingly from the LLA. Overall, dynamic assessment of deviations from the LLA appeared superior to evaluating deviations from the fixed BTF targets. Incorporating LLA metrics into models that include clinical parameters should be tested further to support their diagnostic performance.

Using diverse statistical methods and metrics that incorporate both the intensity and percentage time during which CPP falls below various CPP targets, we highlight the clinical potential of the LLA. Conversely, deviations from CPPopt, as well as from the fixed CPP targets proposed by the Brain Trauma Foundation (BTF), did not convey meaningful prognostic information in our analysis. While explorations assessing autoregulation informed CPP targets in paediatric TBI remain scarce, previous explorations in retrospective datasets with fewer patients have also described the association between CPP below autoregulation informed CPP targets and outcome.^15^, ^16^, ^17^, ^18^ The comparatively lower importance of CPPopt relative to the LLA may be explained by the specific relationship between CPP and autoregulation observed in paediatric patients. Indeed, previous studies examining paediatric TBI have demonstrated that once CPP exceeds the LLA, the association between CPP and PRx remains relatively stable, without substantial increases at higher CPP levels. This suggests that paediatric patients are more markedly affected by low rather than high CPP.^26^^,^^27^

From a clinical perspective, it is important to note that the LLA is both patient specific and dynamically variable over time. The temporal profile of LLA demonstrated that individuals with favourable outcomes exhibited a relatively stable LLA (consistently around 55 mmHg). In contrast, patients with unfavourable outcomes demonstrated significant temporal variability in LLA: initially similar to the favourable group at approximately 55 mmHg on day 0, their LLA subsequently increased, reaching nearly 60 mmHg by day 5. This pattern highlights an increased CPP requirement to maintain adequate cerebral blood flow and suggests the clinical importance of closely monitoring the dynamic behaviour of LLA, especially in patients at risk of unfavourable outcomes.

Current guidelines suggest fixed targets between 40 and 50 mmHg, emphasising that younger patients likely require lower CPP targets compared to adolescents.^1^ In our cohort, the LLA exceeded 50 mmHg during more than 35% of the monitoring time across all patients. Notably, in older children (≥8 years), around 50% of recorded LLA values were above the upper limit of the guideline-recommended threshold of 50 mmHg. Given that LLA was consistently above 50 mmHg even in children with favourable outcome (across all ages), our results suggest that 50 mmHg should be the hard lower limit of CPP in paediatric TBI instead of currently suggested 40 mmHg. However, adequately powered prospective studies are required to identify how continuous LLA-based monitoring should be implemented in paediatric clinical guidelines.

Our analyses are limited to a relatively small sample size (n = 135) with insufficient numbers for age-stratified analyses. Yet, LLA remained a promising target even after correction for the known prognostic parameters. As most research on PRx derived targets has been performed in adult TBI cohorts, the parameters used for estimation of CPPopt and LLA are derived from the adult cohort, adjusted for the paediatric physiology. Whether these represent the optimal parameters remains to be explored. A notable consideration of this analysis is the use of a PRx threshold of 0.2 for LLA calculation. The primary STARSHIP analysis suggested that a PRx threshold of 0 was best for predicting outcome, but mortality-based assessments were not possible owing to low death numbers. When exploring a PRx threshold for LLA estimation, a threshold that reflects *the onset of cerebrovascular autoregulation impairment* is required. Furthermore, the PRx threshold of 0.2 was identified in a large single centre paediatric TBI study where mortality analyses were permitted.^26^ Higher thresholds of 0.25 and 0.3 were also explored, but we opted for 0.2, with the understanding that this threshold would better represent the *onset* of CA impairment.

Importantly, as this study represents a secondary analysis of observational data, we did not have detailed information regarding specific therapies administered to modify CPP. Patient management across the participating centres generally adhered to BTF guidelines, encompassing both CPP and ICP control. Thus, our analysis primarily captures secondary deviations from these standard targets–deviations that were likely unavoidable even under optimal clinical care. Additionally, although CPP can often be augmented through therapeutic interventions, the personalised targets (CPPopt and LLA, both based on PRx application) remain independent of the immediate CPP value, as they reflect the underlying state of cerebral autoregulation. Despite carefully adjusting for various established prognostic covariates, our analysis remains observational and therefore identifies associations rather than causal relationships. Prospective interventional studies are needed to validate the clinical utility of LLA and to determine whether actively targeting LLA leads to improved outcomes or improved prognostication.

## Contributors

All authors had full access to all the data in the study and accept responsibility to submit for publication. All authors read and approved the final version of the manuscript. Shruti Agrawal and Peter Smielewski had access to and verified the underlying data. Specific further contributions were the following. Concept and design: Agrawal, Smielewski, Smith, Bögli; Acquisition of data: Agrawal, Cabeleira, Placek, White, Daubney, Kayani, O'Donnell, Pathan, Krishnan, Bangalore, Sundararajan, Subramanian, Raffaj, Sarfatti, Lampariello, Mayer, Ross; Statistical analysis: Bögli, Smith; Interpretation of the results: Bögli, Smith, Smielewski, Agrawal; Drafting of the manuscript: Bögli, Smith; Critical revision of the manuscript for important intellectual content: All authors; Obtained funding: Agrawal, Smielewski, Czosnyka, Hutchinson; Supervision: Smielewski, Hutchinson, Agrawal.

## Data sharing statement

Postprocessed data is available upon reasonable request to the corresponding author.

## Declaration of interests

**The STARSHIP study** was funded by Action Medical Research for Children's Charity and Addenbrookes Charitable Trust, UK (Grant number–GN2609). Cambridge University Hospitals is the study sponsor (Reference: A094693, contact person: Michelle Ellerbeck–michelle.ellerbeck@nhs.net). The funders or the sponsor did not have any role in the collection, management, analysis, and interpretation of the data; preparation, review, or approval of the manuscript; or decision to submit the manuscript for publication, the right to veto publication or control the decision regarding which journal the manuscript was submitted. **Stefan Yu Bögli** is supported by the Swiss National Science Foundation (SNSF grant number: 225270). **Claudia Ann Smith** is supported by the Patrick & Margaret Flanagan Skye Cambridge Trust Scholarship. **Erta Beqiri** was supported by the Medical Research Council (grant number MR N013433-1) and by the Gates Cambridge Scholarship. **Peter J Hutchinson** is supported by the National Institute for Health Research (NIHR): research professorship, Biomedical Research Centre and Global Neurotrauma Research group and the Royal College of Surgeons of England. This research was supported by the NIHR Cambridge Biomedical Research Centre (NIHR203312∗). The views expressed are those of the authors and not necessarily those of the NIHR or the Department of Health and Social Care. ICM+ is a software licenced by Cambridge Enterprise Ltd. Marek Czosnyka and Peter Smielewski have a financial interest in a part of licencing fee; the licencing fee was waived for this study.

## Appendix A Supplementary data

Supplements A and B

Supplement C_STARSHIP Study Team
