# Supplementary material for: Dynamic versus fixed cerebral perfusion pressure targets in paediatric traumatic brain injury: a STARSHIP analysis
Source: eClinicalMedicine. 2025 Jul 17;86:103370. doi: 10.1016/j.eclinm.2025.103370 (PMC12303001; doi:10.1016/j.eclinm.2025.103370)
Supplement: Supplements A and B [file mmc1.docx]

**Supplement**

Supplement A. **Data coverage.** The data coverage is shown in form of boxplots (length - A), histograms (length per patient - B) and density (frequency of data depending on day post injury with day 0 being the day of the initial injury) (C). The median number of hours available was 157 (IQR 88-279) hours for arterial blood pressure, and 153 (88-275) hours for intracranial pressure respectively. The vast majority was acquired within the first week of injury.


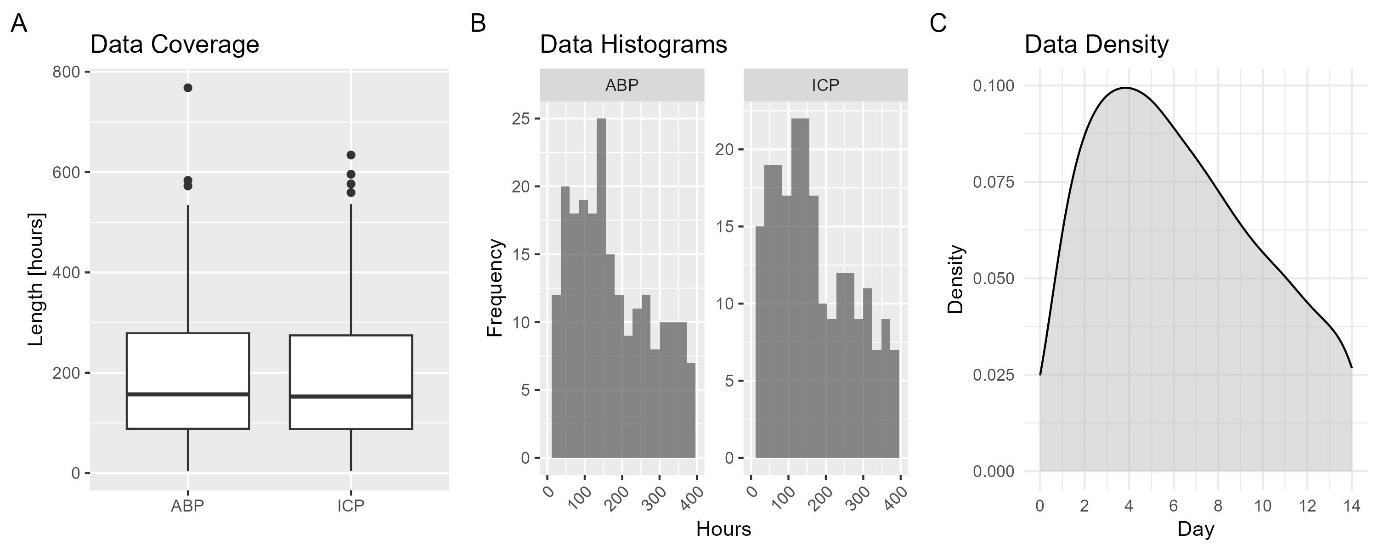


Supplement B. **Sliding Dichotomy – Descriptors Outcome Groups.** The differences in prognostic risk scores are shown stratified by the corresponding risk group using boxplots. The corresponding descriptors are shown in the table below. Distinctly higher disease severities were found for patients with intermediate or high risk for unfavourable outcomes.


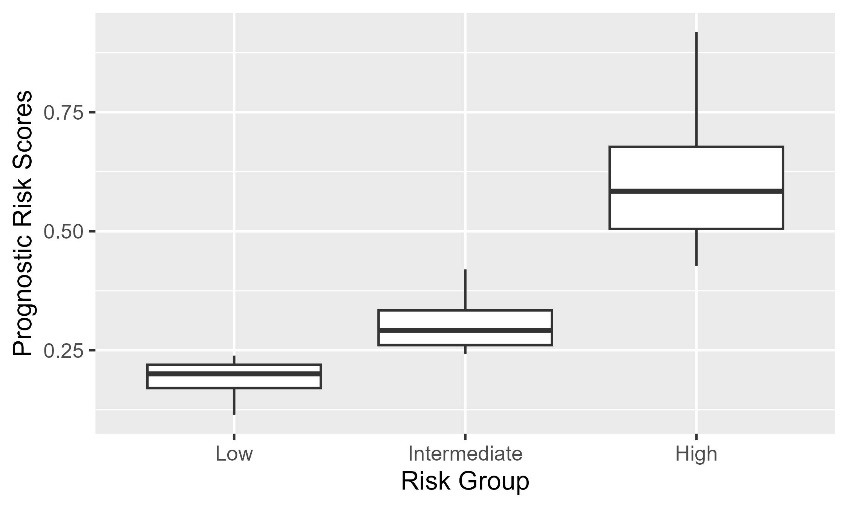


| **Characteristic** | **Low**  N = 39 | **Intermediate**  N = 37 | **High**  N = 39 | **p-value** |
| --- | --- | --- | --- | --- |
| Age (months) | 128 (66, 158) | 130 (43, 156) | 133 (90, 159) | 0.8 |
| Sex |  |  |  | 0.8 |
| Female | 9 (23%) | 10 (27%) | 8 (21%) |  |
| Male | 30 (77%) | 27 (73%) | 31 (79%) |  |
| Type of Injury |  |  |  | >0.9 |
| Blunt | 38 (97%) | 36 (97%) | 38 (97%) |  |
| Penetrating | 1 (2.6%) | 1 (2.7%) | 1 (2.6%) |  |
| Isolated TBI | 22 (56%) | 18 (49%) | 13 (33%) | 0.12 |
| GCS | 7 (4, 11) | 7 (5, 9) | 4 (3, 6) | <0.001 |
| GCS Motor | 4 (1, 5) | 4 (2, 5) | 2 (1, 3) | 0.002 |
| Pupillary Reactivity |  |  |  | <0.001 |
| both reactive | 39 (100%) | 35 (95%) | 19 (49%) |  |
| one reactive | 0 (0%) | 2 (5.4%) | 10 (26%) |  |
| none reactive | 0 (0%) | 0 (0%) | 10 (26%) |  |
| ISS | 26 (22, 29) | 30 (25, 38) | 45 (29, 66) | <0.001 |
| AIS Head | 5 (3, 5) | 5 (4, 5) | 5 (5, 5) | <0.001 |
| Rotterdam Score | 2 (2, 2) | 3 (2, 3) | 3 (3, 4) | <0.001 |
| Hypoxia | 1 (2.6%) | 1 (2.7%) | 15 (38%) | <0.001 |
| Hypotension | 10 (26%) | 3 (8.1%) | 11 (28%) | 0.065 |
