# Supplementary material for: Dynamic versus fixed cerebral perfusion pressure targets in paediatric traumatic brain injury: a STARSHIP analysis
Source: eClinicalMedicine. 2025 Jul 17;86:103370. doi: 10.1016/j.eclinm.2025.103370 (PMC12303001; doi:10.1016/j.eclinm.2025.103370)
Supplement: Supplement C_STARSHIP Study Team [file mmc2.docx]

| **STARSHIP Study Team** | | | | |
| --- | --- | --- | --- | --- |
| **First Name** | **Last Name** | **Institution** | **Location** | **Role** |
| Shruti | Agrawal | Cambridge University Hospitals | Cambridge | Principal Investigator |
| Peter | Smielewski | University of Cambridge | Cambridge | Principal Investigator |
| Peter J. | Hutchinson | Cambridge University Hospitals | Cambridge | Principal Investigator |
| Stefan Yu | Bögli | University of Cambridge | Cambridge | Postdoc Researcher |
| Claudia A. | Smith | University of Cambridge | Cambridge | PhD Candidate |
| Carly | Tooke | Birmingham Children’s Hospital | Birmingham | Research nurse |
| Caroline | Payne | Great Ormond Street Hospital | London | Research nurse |
| Holly | Belfield | Great Ormond Street Hospital | London | Research nurse |
| Amisha | Mistry | Leeds Children’s Hospital | Leeds | Research nurse |
| Collette | Spencer | Leeds Children’s Hospital | Leeds | Research nurse |
| Claire | Jennings | Royal Manchester Children’s Hospital | Manchester | Research nurse |
| Lara | Bunni | Royal Manchester Children’s Hospital | Manchester | Research fellow |
| Laura | Anderson | Nottingham Children’s Hospital | Nottingham | Research nurse |
| Emily | Morgan | Nottingham Children’s Hospital | Nottingham | Research nurse |
| Melanie | James | Oxford University Hospitals | Oxford | Research nurse |
| Rebecca | Beckley | Oxford University Hospitals | Oxford | Research nurse |
| Tahnima | Khatun | Royal London Hospital | London | Research nurse |
| Hafiza | Khatun | Royal London Hospital | London | Research nurse |
| Olivia | Nugent | Royal London Hospital | London | Research nurse |
| Richard | Aldridge | Royal London Hospital | London | PICU technician |
| Ruth | Morgan | Sheffield Children’s Hospital | Sheffield | Research nurse |
| Julie | Morcombe | Sheffield Children’s Hospital | Sheffield | Research nurse |
| Martin | Quinton | Sheffield Children’s Hospital | Sheffield | PICU technician |
| Catherine | Postlethwaite | University Hospitals Southampton | Southampton | Research nurse |
| Jenny | Pond | University Hospitals Southampton | Southampton | Research nurse |
| Jessica | Cutler | University Hospitals Southampton | Southampton | Research nurse |
| Caitlin | Oxford | University Hospitals Southampton | Southampton | Research nurse |
| Marek | Czosnyka | University of Cambridge | Cambridge | Site investigator |
| Michal | Placek | University of Cambridge | Cambridge | Postdoctoral Researcher |
| Manuel | Cabaleira | University of Cambridge | Cambridge | Research Associate |
| Deborah | White | Cambridge University Hospitals | Cambridge | Research nurse |
| Esther | Daubney | Cambridge University Hospitals | Cambridge | Research nurse |
| Adam | Young | University of Cambridge | Cambridge | Site investigator |
| Erta | Beqiri | University of Cambridge | Cambridge | PhD Candidate |
| Riaz | Kayani | Cambridge University Hospitals | Cambridge | Site investigator |
| Roddy | O'Donnell | Cambridge University Hospitals | Cambridge | Site investigator |
| Nazima | Pathan | University of Cambridge | Cambridge | Site investigator |
| Suzanna | Watson | Cambridge Centre for Paediatric Neuropsychological Rehabilitation | Cambridge | Paediatric Neuropsychologist |
| Anna | Maw | Cambridge University Hospitals | Cambridge | Site investigator |
| Matthew | Garnett | Cambridge University Hospitals | Cambridge | Site investigator |
| Hari Krishnan | Kanthimathinathan | Birmingham Children’s Hospital | Birmingham | Site investigator |
| Harish | Bangalore | Great Ormond Street Hospital | London | Site investigator |
| Santosh | Sundararajan | Leeds Children’s Hospital | Leeds | Site investigator |
| Gayathri | Subramanian | Royal Manchester Children’s Hospital | Manchester | Site investigator |
| Dusan | Raffaj | Nottingham Children’s Hospital | Nottingham | Site investigator |
| Simona | Lampariello | Oxford University Hospitals | Oxford | Site investigator |
| Avishay | Sarfatti | Royal London Hospital | London | Site investigator |
| Anton | Mayer | Sheffield Children’s Hospital | Sheffield | Site investigator |
| Oliver | Ross | Southampton General Hospital | Southampton | Site investigator |
